# Genome-wide transcriptional adaptation to salt stress in *Populus*

**DOI:** 10.1186/s12870-019-1952-2

**Published:** 2019-08-20

**Authors:** Jin-Gui Liu, Xiao Han, Tong Yang, Wen-Hui Cui, Ai-Min Wu, Chun-Xiang Fu, Bai-Chen Wang, Li-Jun Liu

**Affiliations:** 1 State Forestry and Grassland Administration Key Laboratory of Silviculture in downstream areas of the Yellow River, College of Forestry, Shandong Agriculture University, Taian, 271018 Shandong China; 2 State Key Laboratory of Subtropical Silviculture, College of Forestry and Biotechnology, Zhejiang A&F University, Lin’an, Hangzhou, 311300 China; 3 Guangdong Key Laboratory for Innovative Development and Utilization of Forest Plant Germplasm, College of Forestry and Landscape Architecture, South China Agricultural University, Guangzhou, 510642 China; 4 Key Laboratory of Biofuels, Qingdao Engineering Research Center of Biomass Resources and Environment, Qingdao Institute of Bioenergy and Bioprocess Technology, Chinese Academy of Sciences, Qingdao, 266101 Shandong China; 5 Photosynthesis Research Center, Key Laboratory of Photobiology, Institute of Botany, Chinese Academy of Sciences, Beijing, 100093 China

**Keywords:** Abiotic stress, Adaptation, Perennial plants, *Populus*, Gene module

## Abstract

**Background:**

Adaptation to abiotic stresses is crucial for the survival of perennial plants in a natural environment. However, very little is known about the underlying mechanisms. Here, we adopted a liquid culture system to investigate plant adaptation to repeated salt stress in *Populus* trees.

**Results:**

We first evaluated phenotypic responses and found that plants exhibit better stress tolerance after pre-treatment of salt stress. Time-course RNA sequencing (RNA-seq) was then performed to profile changes in gene expression over 12 h of salt treatments. Analysis of differentially expressed genes (DEGs) indicated that significant transcriptional reprogramming and adaptation to repeated salt treatment occurred. Clustering analysis identified two modules of co-expressed genes that were potentially critical for repeated salt stress adaptation, and one key module for salt stress response in general. Gene Ontology (GO) enrichment analysis identified pathways including hormone signaling, cell wall biosynthesis and modification, negative regulation of growth, and epigenetic regulation to be highly enriched in these gene modules.

**Conclusions:**

This study illustrates phenotypic and transcriptional adaptation of *Populus* trees to salt stress, revealing novel gene modules which are potentially critical for responding and adapting to salt stress.

**Electronic supplementary material:**

The online version of this article (10.1186/s12870-019-1952-2) contains supplementary material, which is available to authorized users.

## Background

Adaptation to various abiotic stresses is critical for the survival and biomass accumulation of sessile plants and is particularly true for perennial tree species due to their relatively long-life cycle. *Populus*, a model tree species due to the availability of a near complete set of experimental resources such as easy propagation, transformation methods, and abundance of genetic and genomic materials [1, 2], provides an ideal system to uncover how perennial trees adapt to abiotic stresses.

Recent studies in *Arabidopsis thaliana* and other model plants show that plants experiencing sub-lethal abiotic stress may memorize that stress at physiological and transcriptional levels to promote better performance when they encounter the same stress again [3–8]. For example, Arabidopsis and maize plants that have experienced one or more cycles of dehydration stress and watered recovery exhibit greater ability to retain leaf relative water content (RWC) compared to plants that have no experience of dehydration [9–11]. At the transcriptional level, stress response genes can be divided into two types based on their responsiveness to successive stress: ‘memory genes’ that show significantly different levels of up- or down-regulation in subsequent stress than the previous one, and ‘non-memory genes’ that show similar responses to each stress [12]. Comparative studies between Arabidopsis, maize, and switchgrass found that there are conserved dehydration memory genes but also remarkable differences in the total number and homologs of dehydration memory genes [10, 13], suggesting the existence of both evolutionarily conserved and species-specific mechanisms regulating plant responses to repeated abiotic stresses.

Transcriptional regulation is dynamic, and different types of genes usually respond to perturbations with different kinetics and patterns. Thus, it would be much more informative to profile plant transcriptional responses to abiotic stress with multiple time points of gene expression data rather than with a single time point [14–16]. The increasing capacity of high-throughput sequencing makes it feasible to characterize whole genome transcriptional dynamics with time-series RNA-seq experiments. Coupled with computational analysis, it is possible to identify key gene modules, hub genes or infer the hierarchical structure of the regulatory network using this kind of time-series expression data [16–23], and provide a better overview of how underlying biological processes are regulated.

High salinity, usually presented by accumulation of NaCl in soil, causes osmotic and ionic stresses, and is one of the most widely spread abiotic stresses that limit plant growth and distribution [24, 25]. Therefore, it is practically important to investigate the mechanisms of how plants adapt to salt stress. In this study, we established a precisely controlled liquid culture-based experimental procedure to investigate plant adaptation to salt stress in *Populus*. We first evaluated the phenotypic responses to salt stress and then performed time-course RNA-seq to characterize transcriptome dynamics during salt treatments. Our results showed that *Populus* plants displayed quick adaptation to salt stress phenotypically and transcriptionally. Key gene modules were identified through co-expression analysis, and the biological relevance of these gene modules were analyzed.

## Results

### Phenotypic adaptation to salt stress in *Populus*

**Fig. 1:**
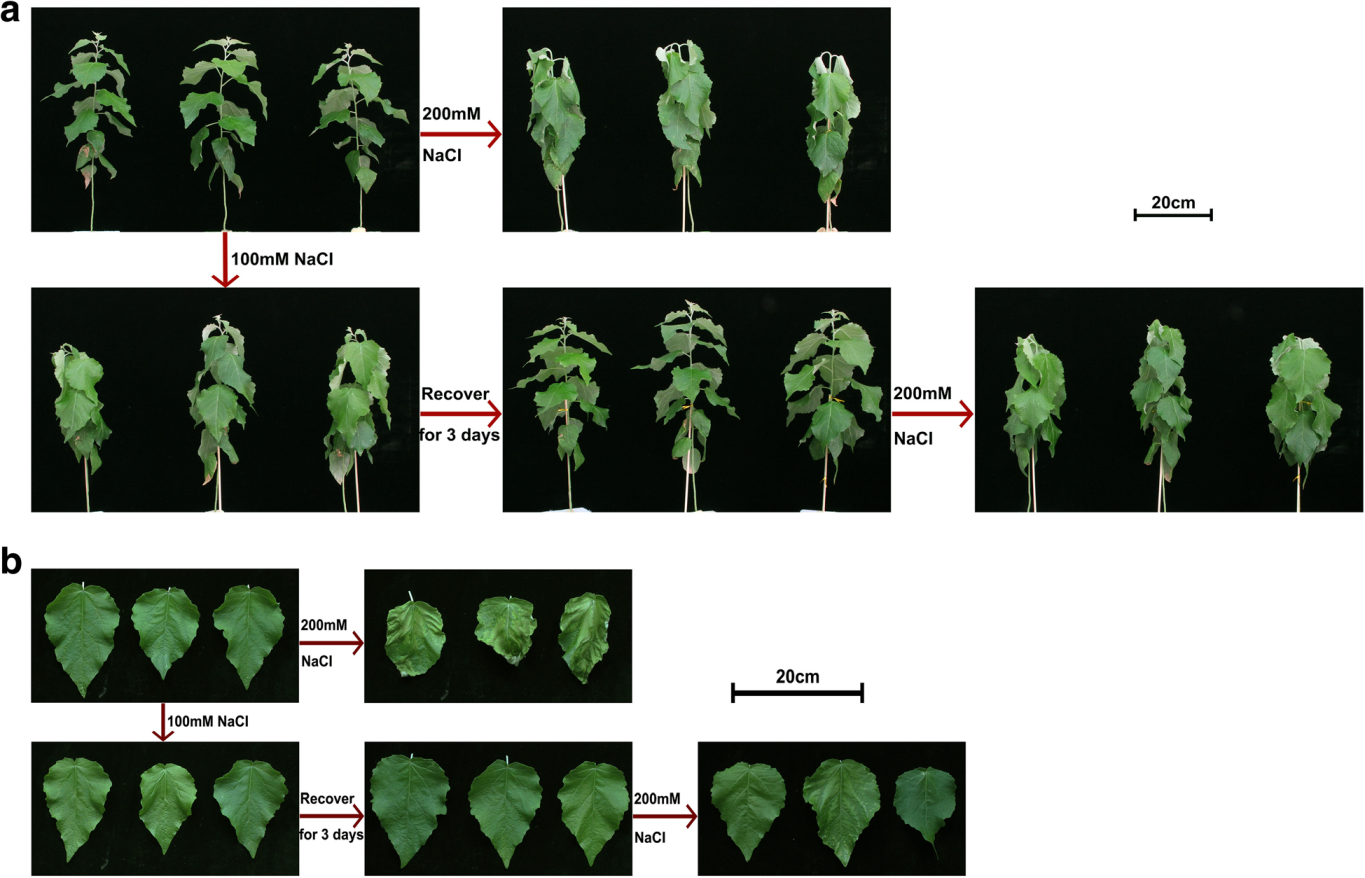
Plant phenotypic responses during repeated NaCl treatments in *Populus*. **a** Whole plants responses to salt stress. Photos were taken 1 h after each NaCl treatment. Scale bar, 20 cm. **b** Leaf phenotype during salt stress. Photos were taken 24 h after each NaCl treatment. Scale bar, 20 cm

In order to study plant adaptation to salt stress, we adopted the liquid culture system of *Populus* that allows precisely controlled NaCl treatments. We set up two groups of experiments: for the first group, we used 200 mM NaCl to do a high salt treatment directly; for the second group, we used 100 mM NaCl to do 1 day low salt treatment, then followed by 3 days of recovery culture, and finally high salt treatment with 200 mM NaCl. In the first group, plants quickly showed a significantly severe stress phenotype, with strong shoot apical bend and leaves dropping. By contrast, in the second group, plants treated with low salt acted similar to the control plants, indistinguishable in phenotype to the control plants after 3 days of recovery and showed a mild response following 200 mM NaCl salt treatment (Fig. 1a). Time-course analysis further supported improved plant tolerance to NaCl treatment in the second group: plants responded to 200 mM NaCl treatment after 0.5 h and started to recover after 6 h while plants directly treated with 200 mM NaCl showed a much stronger phenotype after 0.5 h and started to recover after 12 h (Additional file 1: Figure S1). Detailed inspection of the leaf phenotype showed consistent results with whole plant responses (Fig. 1b), with severe leaf damage in the first group, and mild damage in the second group. Overall, these results indicated that *Populus* plants could physiologically adapt to salt stress quickly after a pre-treatment.

### RNA-seq reveals massive transcriptional reprogramming in repeated salt stress

**Fig. 2:**
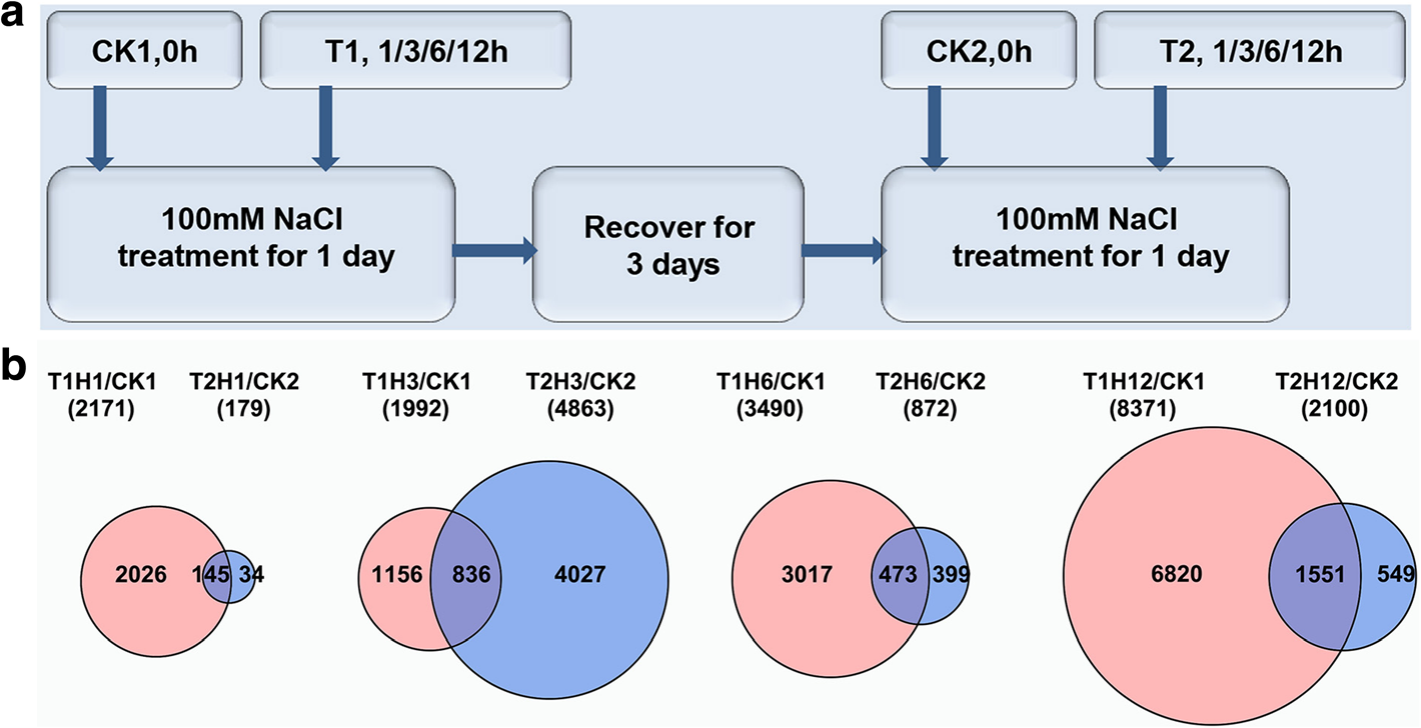
Overview of the RNA-seq data. **a** Workflow for RNA-seq sample preparation. **b** Venn diagrams show overlap of differentially expressed genes (DEGs) between the same time point of repeated NaCl treatment. T1: the first salt treatment; T2: the second salt treatment after 3 days recovery following the first salt treatment; H1/H3/H6/H12: 1/3/6/12 h of salt treatment

We next performed RNA-seq to profile the dynamic changes of genome-wide transcript abundance during repeated salt treatments (Methods). We first treated the plants with 100 mM NaCl for 1 day, followed by 3 days of recovery culture, and then repeated 100 mM NaCl treatment again. Samples were collected after 0 h (control samples, designated as CK1 and CK2, respectively), 1 h, 3 h, 6 h, and 12 h (designated as T1H1, T1H3, T1H6, T1H12, T2H1, T2H3, T2H6, and T2H12, respectively) of each salt treatment (Fig. 2a). Notably, for RNA-seq sample preparation, we used 100 mM NaCl for both salt treatments to avoid the influence of different NaCl concentrations on transcription, and all time points for each treatment were collected within 2 h to minimize the influences of circadian rhythms or other environmental factors. High quality total RNAs were isolated and submitted for RNA-seq library construction and sequencing. At least 45–76 million 150 bp paired-end clean reads were obtained for each library (Additional file 7: Table S1).

To identify differentially expressed genes (DEGs) during each salt treatment, we performed pair-wise edgeR analysis between individual time point and the corresponding control samples. The number of DEGs changed dramatically along the time-course of each treatment (Additional file 8: Table S2; Additional file 9: Table S3). Moreover, DEGs at the same time point of the first treatment (T1) and the second treatment (T2) were dramatically different. For example, there were 2171 DEGs in the T1H1/CK1 group whereas there were only 179 in the T2H1/CK2 group. Overlapping studies found that there were 145, 836, 473, and 1551 common genes for each time point (Fig. 2b), which account for 81, 41, 54, and 73% of the smaller dataset, respectively. Overall, the differences in the total number of DEGs and the low overlapping rates indicate that massive transcriptional reprogramming during repeated salt treatments occurred. Notably, there were 1051 DEGs between CK1 and CK2, with 649 up-regulated and 402 down-regulated DEGs. GO function annotation showed that 43 out of 1051 DEGs were involved in response to salt stress. Among those 43 DEGs, 19 were up-regulated, including membrane transporter proteins and MYB transcription factors; 24 were down-regulated, including tonoplast intrinsic protein TIP1, drought-induced protein Di19, gibberellin-regulated protein GASA14, and calcium signaling proteins CDPK and GAP1 (Additional file 9: Table S3).

### Co-expression analysis identifies key gene modules in response to repeated salt stress

**Fig. 3:**
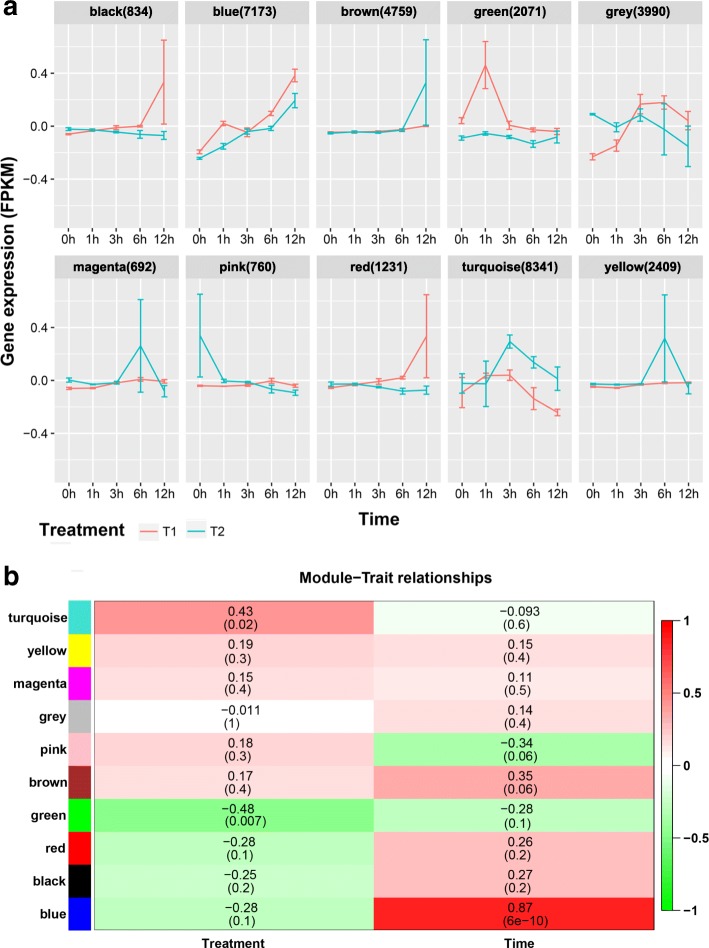
Co-expressed gene modules identified by Weighted Gene Co-expression Network Analysis (WGCNA). **a** Module dynamic eigengene expression in response to NaCl treatments (T1, T2). The numbers in parentheses represent the number of genes in the module. **b** Correlation of individual gene module with Treatment (T1, T2) and Time (0 h, 1 h, 3 h, 6 h, 12 h). The number stand for correlation value and related *P* value (in parentheses) between the module and “Treatment” or “Time”. *P* value < 0.05 was the statistical significance threshold

To better understand the correlation of gene expression between time-course treatments, weighted gene correlation network analysis (WGCNA) was performed. In total, 10 co-expressed gene modules, which showed different expression curves and peaks during NaCl treatments, were identified (Fig. 3a; Additional file 2: Figure S2; Additional file 10: Table S4). Module size ranged from 692 to 8341 genes, with the green module containing a moderate amount (2071 genes), while the blue (7173 genes) and turquoise (8341 genes) modules contained the largest number of genes (these three modules showed significant correlation with salt stress response or adaptation as discussed later) (Table 1).

**Table 1:** Summary of all co-expression gene modules

Modules	Gene number^a^	TF number^b^	% of TF^c^	*P*-value^d^
Magenta	692	64	9.25	0.000119395
Pink	760	29	3.82	0.002390257
Black	834	52	6.24	0.05321326
Red	1231	58	4.71	0.009453082
Green	2071	196	9.46	1.66995E-11
Yellow	2409	182	7.56	9.83444E-05
Grey	3990	270	6.77	0.001601634
Brown	4759	345	7.25	6.59708E-06
Blue	7173	517	7.21	7.64648E-08
Turquoise	8341	376	4.51	7.21849E-11

*TF* Transcription Factor

^a^gene numbers in each module, ^b^TF numbers in each module, ^c^% of TF genes in each module, ^d^*P*-value was the statistical result of number of TF gene in the corresponding gene module and was derived from HYMGEOMDIST test. % of TFs in *Populus* genome is 5.91%

Gene expression profile was visualized with eigengene values (the first principal component of transcript profiles) for each module and showed distinct co-expression patterns across modules (Fig. 3a): the blue module increased along the time-course and showed similar changes between the first and the second treatment, the green module showed a significant peak at 1 h of the first treatment compared to the second treatment, and the turquoise module showed a significant peak at 3 h of the second treatment compared to the first treatment, the other seven modules did not have significant changes across the time-course. Consistently, Module-Trait relationships showed that the green and turquoise modules highly correlated with “Treatment” (T1 and T2), whereas the blue module significantly correlated with “Time” (1 h, 3 h, 6 h, and 12 h) (Fig. 3b). Other seven modules showed relatively low correlation to any variable. These results indicated that the green and turquoise modules may be critical for adaptation to repeated salt stress, whereas the blue module appears to be essential for each salt stress response. Therefore, we focused on analyzing the green, turquoise, and blue modules.

The salt overly sensitive (SOS) pathway is essential for salt tolerance in plants [26]. There are three homologs of SOS1, two of SOS2, and five of SOS3 in *Populus* genome. We checked our co-expression analysis and found that two homologs of SOS1 were classified into turquoise module, two of SOS2 were classified into blue module, and one of the SOS3 was in green module and one was in turquoise module (Additional file 10: Table S4).

Transcription factors (TFs) are important regulators in plant development and response to stress [27–33]. Therefore, we investigated the distribution of TF genes in these three gene modules. Compared to the whole genome, the green and blue modules contained a significantly higher percentage of TF, whereas the turquoise module contained a significantly lower percentage of TF (Table 1; Additional file 11: Table S5). Additional file 12: Table S6 shows a summary of individual TF families in the green, turquoise, and blue modules. Statistical analysis found that the green module was significantly enriched in the MYB/MYB-related, ERF, bHLH, and HD-ZIP families of TFs, whereas the blue module was significantly enriched in the NAC, bZIP, WRKY, C3H, B3 and HSF families of TFs. These results provide valuable information for identifying key regulators that underly plant responses to salt stress in general, but more importantly, adaptation to salt stress.

### Gene ontology enrichment analysis of the key gene modules

To further explore the functional significance of the key gene modules, we performed gene ontology (GO) enrichment analysis to identify significantly enriched biological pathway (BP), molecular function (MF), and cellular component (CC) GO terms of each gene module. In total, 399, 154, and 681 enriched BP GO terms were identified from the blue, green, and turquoise modules, respectively (Additional file 13: Table S7).

Plant hormones are important regulators for abiotic stress responses. In this study, we found enriched GO terms for all classical plant hormone pathways in the selected gene modules (Table 2). Interestingly, genes functioning in auxin polar transport and signaling are mainly enriched in the green and turquoise modules, whereas genes functioning in abscisic acid (ABA) and salicylic acid (SA) signaling pathways are only enriched in the blue module. Jasmonic acid (JA) signaling pathways are highly enriched in the blue module. Genes functioning in responses to plant hormone gibberellins (GA), brassinosteroid (BR), and ethylene (ET) are also enriched in the green module. These results indicate that plant hormones, especially auxin may be critical for plant adaptation to repeated salt stress while ABA, SA, and JA are essential for plant responses but not sufficient for plant adaptation to each salt stress.

**Table 2:** Enrichment of plant hormone and cell wall related gene ontology (GO) categories in each gene module

GOBPID	*P*-value	Module	Term
Auxin			
GO:0009734	1.38E-07	green	auxin-activated signaling pathway
GO:0009733	3.62E-07	green	response to auxin
GO:0071365	4.58E-07	green	cellular response to auxin stimulus
GO:0060918	0.001810948	green	auxin transport
GO:0009926	0.002255868	green	auxin polar transport
GO:0009926	0.006351523	pink	auxin polar transport
GO:0060918	0.008487031	pink	auxin transport
GO:0071365	0.000658331	turquoise	cellular response to auxin stimulus
GO:0009734	0.003241714	turquoise	auxin-activated signaling pathway
GO:0010540	0.00491535	turquoise	basipetal auxin transport
GO:0009733	0.00886687	turquoise	response to auxin
Gibberellins			
GO:0010371	0.006713667	brown	regulation of gibberellin biosynthetic process
GO:0009739	0.000443904	green	response to gibberellin
Cytokinin			
GO:0080037	0.001227355	blue	negative regulation of cytokinin-activated signaling pathway
GO:0009735	0.000982346	turquoise	response to cytokinin
Brassinosteroid			
GO:0009741	0.00057973	green	response to brassinosteroid
Ethylene			
GO:0009723	4.79E-05	black	response to ethylene
GO:0009873	0.000328341	black	ethylene-activated signaling pathway
GO:0071369	0.000505744	black	cellular response to ethylene stimulus
GO:0071369	4.95E-05	green	cellular response to ethylene stimulus
GO:0009873	0.000260229	green	ethylene-activated signaling pathway
GO:0009723	0.000263073	green	response to ethylene
GO:0010105	3.66E-07	red	negative regulation of ethylene-activated signaling pathway
GO:0010104	6.91E-06	red	regulation of ethylene-activated signaling pathway
Jasmonic acid			
GO:0009753	0.000493563	blue	response to jasmonic acid
GO:2000022	0.001852248	blue	regulation of jasmonic acid mediated signaling pathway
GO:0009867	0.002393932	blue	jasmonic acid mediated signaling pathway
GO:0071395	0.00350308	blue	cellular response to jasmonic acid stimulus
GO:0009694	0.000172051	green	jasmonic acid metabolic process
GO:0009753	0.001153948	green	response to jasmonic acid
Abscisic acid			
GO:0009737	4.22E-06	blue	response to abscisic acid
GO:0009738	0.00056742	blue	abscisic acid-activated signaling pathway
GO:0071215	0.003692747	blue	cellular response to abscisic acid stimulus
Salicylic acid			
GO:0009862	6.20E-07	blue	systemic acquired resistance, salicylic acid mediated signaling pathway
GO:0010337	0.001421978	blue	regulation of salicylic acid metabolic process
GO:0009863	0.001776784	blue	salicylic acid mediated signaling pathway
GO:0071446	0.002641471	blue	cellular response to salicylic acid stimulus
GO:0009751	0.004901824	blue	response to salicylic acid
GO:0046244	0.005445888	blue	salicylic acid catabolic process
Cell wall			
GO:0044036	0.00034442	blue	cell wall macromolecule metabolic process
GO:0010383	0.001016966	blue	cell wall polysaccharide metabolic process
GO:0071554	1.66E-07	green	cell wall organization or biogenesis
GO:0042546	2.02E-06	green	cell wall biogenesis
GO:0009834	5.04E-06	green	plant-type secondary cell wall biogenesis
GO:0071669	8.26E-06	green	plant-type cell wall organization or biogenesis
GO:0009832	0.000110576	green	plant-type cell wall biogenesis
GO:1901347	0.000132263	green	negative regulation of secondary cell wall biogenesis
GO:1903339	0.000132263	green	negative regulation of cell wall organization or biogenesis
GO:0042545	0.000240471	green	cell wall modification
GO:0009827	0.000539041	green	plant-type cell wall modification
GO:0071555	0.002039367	green	cell wall organization
GO:0009664	0.003032875	green	plant-type cell wall organization
GO:0009828	0.006460053	green	plant-type cell wall loosening
GO:0016998	0.004935135	pink	cell wall macromolecule catabolic process
GO:0009664	0.008475018	red	plant-type cell wall organization
GO:0071555	0.008547578	red	cell wall organization
GO:0009833	0.000480169	turquoise	plant-type primary cell wall biogenesis
GO:2000652	0.002143591	yellow	regulation of secondary cell wall biogenesis
GO:0009809	2.82E-07	green	lignin biosynthetic process
GO:0009808	4.08E-07	green	lignin metabolic process
GO:0046274	1.47E-05	green	lignin catabolic process
GO:0010410	0.008590021	blue	hemicellulose metabolic process
GO:0045490	0.000254293	green	pectin catabolic process
GO:0045488	0.001028476	green	pectin metabolic process

Detailed analysis confirmed that each module has specific enriched GO terms. For example, the green module showed enrichment in negative regulation of growth (Additional file 3: Figure S3; Additional file 13: Table S7); the turquoise module is enriched in GO terms involved in RNA transport and catabolic, protein modification and transport, and meristem development (Additional file 4: Figure S4; Additional file 13: Table S7); and the blue module is enriched in regulation of mitotic cell cycle, protein dephosphorylation, programmed cell death, and response to abiotic stress (Additional file 5: Figure S5; Additional file 13: Table S7). Remarkably, cell wall related GO terms are highly enriched in green module, such as plant-type cell wall biogenesis, plant-type cell wall modification, cell wall loosening, and lignin/pectin metabolic or catabolic process (Table 2); and the turquoise module is significantly enriched in epigenetic modification pathways such as histone methylation, DNA methylation, and chromatin organization (Additional file 4: Figure S4; Additional file 13: Table S7).

### Verification of gene expression profile of the green, turquoise and blue module

**Fig. 4:**
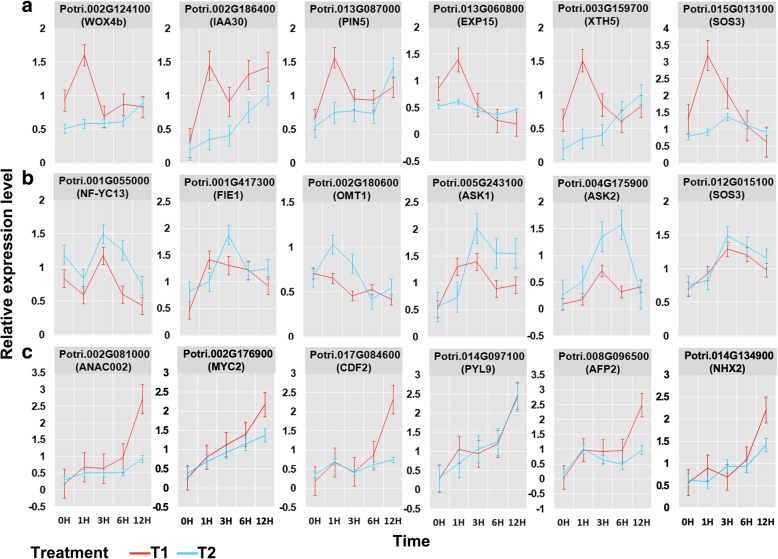
Verification of the expression profile of representative genes from green (**a**), turquoise (**b**), and blue (**c**) module by quantitative real-time PCR. Relative gene expression level was calculated using actin as the internal control. The results were means ± SE with three biological replicates for each sample

To validate the gene expression profiles derived from RNA-seq analysis, we randomly selected six genes from green, turquoise, and blue module with relatively higher module membership value (MM value) and expression level for quantitative PCR (qPCR) test. Similar expression patterns were found for all selected genes (Fig. 4; Additional file 6: Figure S6), suggesting that our results of co-expression analysis with RNA-seq data are reliable for analyzing gene expression in response to repeated salt stress in *Populus*.

### Co-expression network exploration

**Fig. 5:**
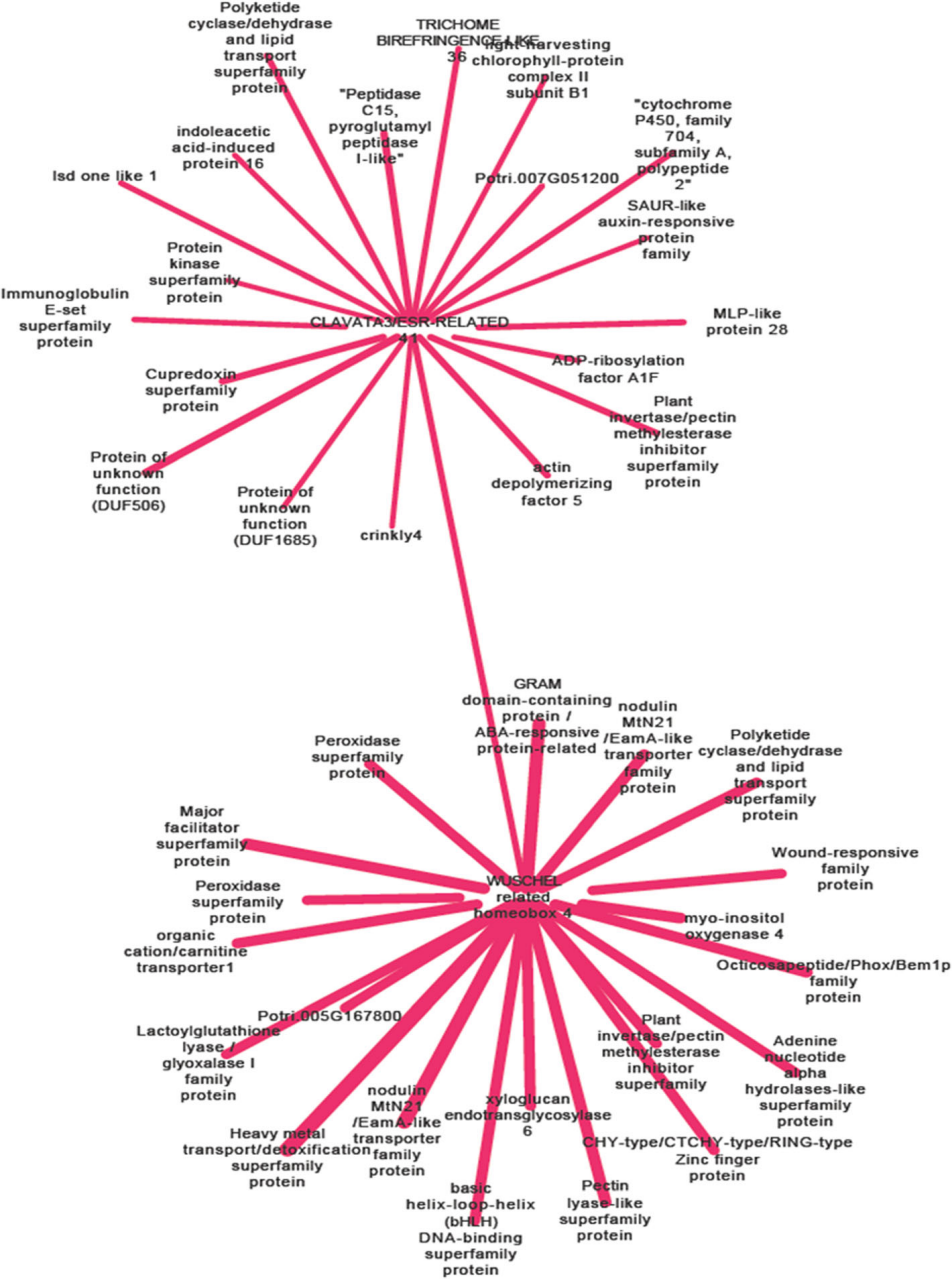
Illustration of network study with our co-expression data, using *WOX4b* and *CLE41a* genes from green module as an example and displaying their top 20 co-expressed neighbor genes

Co-expression network analysis shows the correlation of genes based on their expression patterns, showing possible causality in expression changes of co-expressed genes under certain experimental conditions. *WOX4-CLE41* signaling pathway has been shown as the central regulator of cambium cell fate controlling secondary growth rate in *Populus* [34, 35], therefore, we used the *WOX4b* and *CLE41a* genes from green module as an example to further explore the co-expression network derived from this study. Top 20 co-expressed genes were displayed for each gene (Fig. 5; Additional file 14: Table S8). In *WOX4b* co-expressed genes, *MIOX4*, *bHLH* TF, two peroxidases, several kinds of transporter genes, and cell wall biogenesis and lignification related genes such as pectin lyase-like superfamily protein, plant invertase/pectin methylesterase inhibitor superfamily, and xyloglucan endoxyloglucan transferase *XTH6* were found. Among *CLE41a* co-expressed genes, actin depolymerizing factor *ADF5*, *LOL1*, *MLP28*, gene encoding peptidase C15, protein kinase, and notably, *WOX4b* were found. Moreover, *CLE41a* was reported to be regulated by auxin and there was one SAUR-like auxin-responsive gene and *IAA16* among the *CLE41a* top 20 co-expressed genes. These co-expressed genes are possibly important for the regulation of plant growth during salt stress.

## Discussion

Transcriptional regulation has been shown to play important roles in plant response and adaptation to abiotic stresses [5, 36, 37]. Therefore, identifying key transcriptional regulatory genes and signaling pathways that participate in the regulation of plant response to abiotic stresses is critical for genetic or biotechnological improvement of plant survival rate and biomass production. Recently, there have been several comprehensive studies on plant adaptation to abiotic stresses. However, our knowledge of how perennial trees respond to repeated abiotic stress is still limited. In this study, we used hybrid poplar clone 84 K (*Populus alba* X *Populus glandulosa*) as a model system to investigate plant responses to repeated salt stress, and demonstrated the adaption of perennial trees to abiotic stresses.

### *Populus* plant could adapt to salt stress quickly at both physiological and transcriptional levels

We developed a liquid-culture system, which enabled us to precisely control the NaCl dosage and time of treatments, to investigate how *Populus* plant’s responds to repeated salt stress. Observation of whole plant responses and leaf phenotype to repeated salt stress demonstrated that plants experiencing 1 day of low NaCl pre-treatment followed by 3 days recovery perform much better in high NaCl treatment than plants treated directly with high NaCl (Fig. 1). This suggests *Populus* could adapt to salt stress quickly at the physiological level.

We also performed time-course RNA-seq to profile transcriptional dynamics in response to NaCl treatments, which provide a better resolution than single time point RNA-seq data. The total number of DEGs over the time-course and DEGs pair-wise overlapping study revealed that there were dynamic and massive transcriptional reprogramming in successive salt treatments (Fig. 2; Additional file 8: Table S2). Notably, there were less DEGs in T2 at all time points except 3 h, in which T2H3/CK2 has more DEGs than T1H3/CK1. Furthermore, the turquoise module from our co-expression analysis, which was the largest module and contain 8341 genes, has a relatively higher expression peak in T2H3 than T1H3. The turquoise module was enriched in GO categories such as auxin polar transport and signaling, RNA transport and catabolic, protein modification and transport, meristem development, and epigenetic modification pathways that were possibly important for salt adaptation. Therefore, we thought this could be reflective of plant response to repeated salt stress. Another interesting point from DEG analysis was that, there were still over 1000 genes that showed significantly differential expression after 3 days recovery culture (Additional file 8: Table S2) even though plant phenotype returned to pre-treated status, suggesting that transcriptional regulation is more sensitive than physiological regulation and 3 days recovery culture is not enough to completely reverse the transcriptional changes. This phenomena is consistent with previous results reported by others that plants retains the transcriptional memory of abiotic stress for up to 5 days [8, 9]. It would be interesting to investigate how their transcriptional levels are retained during the recovery period.

### Co-expression analysis identify putative gene modules critical for salt stress adaptation

With this time-series RNA-seq data, gene co-expression analysis was performed using the WGCNA package and 10 modules were identified. Each module has different gene expression profiles along NaCl treatments. Based on the analysis of module-trait correlation we decided to focus on the green, turquoise, and blue modules which showed significantly high correlation to repeated “Treatment” or “Time” (Fig. 3). Overall, genes in the green module reached their expression peak at 1 h of the first treatment but not the second treatment, whereas genes in the turquoise module showed an expression peak at 3 h of the second treatment but not the first treatment. This suggested that genes from these two modules could discriminate the first and repeated salt stresses and possibly lead to salt stress adaptation. On the other hand, expression levels of genes in the blue module increased along the time-course and showed similar patterns between the first and the second salt treatment, indicating that genes from this module are important for the plant response to salt stress in general.

Study of genome-wide TF distribution found that the green and blue modules contained significantly more TF genes whereas the turquoise module contained significantly less, indicating that the green and blue modules may act at a higher hierarchical level of transcriptional regulation that initiates a transcriptional signaling cascade to regulate downstream gene expression. The green module is significantly enriched in MYB/MYB-related, ERF, bHLH, and HD-ZIP families of TFs and the blue module is significantly enriched in NAC, bZIP, WRKY, C3H, B3 and HSF families of TFs. All of these TF families have been shown to be important regulators of abiotic stress responses [27, 28, 30–33, 38, 39]. However, whether they are participating in the regulation of stress adaptation still needs further investigation. This analysis provides putative candidate genes for genetic studies of plant transcriptional responses to abiotic stress for future studies.

Maintaining ion homeostasis is crucial for plant tolerance and adaptation to salt stress. The salt overly sensitive (SOS) pathway, including SOS1, SOS2, and SOS3, has been shown as a central player in sodium ion efflux under salt stress [40–42], and is conserved across different plant species such as Arabidopsis [26], rice [43], tomato [44], and poplar [45]. In our co-expression analysis, two homologs of SOS1 were classified into turquoise module, two of SOS2 were classified into blue module, one of SOS3 was in green module and one was in turquoise module (Fig. 4; Additional file 10: Table S4). The H^+^ electrochemical gradient across membranes could drive the Na^+^ compartmentalization into vacuoles or exclusion out cells, and is generated by H^+^ pump such as H^+^-pyrophosphate VP1 [46]. Two *VP1* homolog genes were belong to turquoise module in our co-expression analysis (Additional file 10: Table S4), one of which have been functionally characterized in poplar and could improve plant growth under salt stress [47]. Together, these results suggest that *Populus* tree may adapt to repeated salt stress through modifying ion sensing and transporting pathways.

### Functional analysis of the gene modules suggested plant hormones and epigenetic modifications are critical for *Populus* plant’s adaptation to salt stress

Plant hormones are important regulators of plant responses to abiotic stress and complex crosstalk occurs among the signaling pathways of different hormones for coordinating plant growth [29, 41, 48–52]. In general, ABA, SA, JA, and ET are characterized as “stress hormones” which are responsible for a plants’ quick response to biotic or abiotic stresses, whereas auxin, GA, BR, and cytokinin are recognized as “growth promoting hormones” which modify plant developmental processes in the long-term [49, 53]. Interestingly, our GO enrichment analysis with co-expression gene modules suggested that plant hormones play important roles in *Populus* response and adaptation to repeated salt stress. Moreover, the above two types of plant hormones may function at different aspects of plant response and adaptation to abiotic stresses. For example, we found that the ABA signaling pathway was only enriched in the blue module, suggesting that ABA signaling is necessary for plant response but not sufficient for plant adaptation to repeated salt stress, which is consistent with previous reports [9, 54]. JA and SA signaling pathways were also specifically enriched in the blue module. On the other hand, auxin, GA, and BR signaling or transport pathways were highly enriched in the green or turquoise modules, which indicated that these plant hormones may play critical roles in plant adaptation to salt stress through modulating the plant growth pattern in the long-term. There are also highly enriched GO terms referring to negative regulation of growth and cell wall biogenesis/modifications in the green module. It would be important to analyze how these plant hormones change and identify their crosstalk signaling pathways in the plant response and adaptation to abiotic stress, thus uncovering key signaling genes for improving plant growth during abiotic stresses.

Epigenetic regulation has been proposed as one of the mechanisms underlying transcriptional stress memory [37, 55–58]. For example, histone trimethylation markers H3K4me3 and H3K27me3 correlate to the target gene’s transcriptional memory in dehydration stress [9, 59, 60]. Also, DNA methylation changes in particular regions of the *Arabidopsis* genome are responsible for hyperosmotic stress memory caused by salt treatments [61]. In our results, we found epigenetic pathways, such as histone methylation, histone ubiquitination, DNA methylation, and chromatin organization, were highly enriched in the turquoise module which correlated with “Treatment” and showed a peak expression level in T2H3 (Fig. 3). To further reveal the mechanisms of plant adaptation to abiotic stress, it would be important to investigate when do these epigenetic modifications establish, the effects of these epigenetic modification on the expression of target genes, and their dynamics during the recovery period.

## Conclusions

In this study, we reported that *Populus* plants could adapt to salt stress quickly in both physiological and transcriptional levels. We also did time-series RNA-seq to profile the transcriptome dynamics during repeated salt treatments and showed that there was significant reprogramming at the transcriptional level. Our co-expression analysis with these time-series RNA-seq data identified 10 co-expressed gene modules, including two modules which were highly correlated to repeated salt stress and one module which was highly correlated to general salt stress response. Gene ontology (GO) analysis suggested that plant hormones regulating plant growth, particularly auxin signaling pathway, are possibly play critical roles in plant adaptation to repeated salt stress, and ABA signaling is critical for general response to salt stress. Cell wall biosynthesis/modification and epigenetic regulation are also suggested as important regulatory pathways for plant adaptation to salt stress. In summary, our results provided a framework for dissecting signaling pathways and identifying key regulatory genes for plant adaptation to salt stress in *Populus*.

## Methods

### Plant cultivation and sample collection

Hybrid poplar (*Populus alba* X *Populus glandulosa*) clone 84 K, grown under 16 h light/8 h dark photoperiod condition and at 25 °C, was used for all experiments. All plants were propagated in Shandong Agriculture University (Taian, Shandong, China). 84 K seedlings were subcultured in magenta boxes under controlled growth chamber, and then transferred to soil 1 month later, finally transferred to full Hoagland nutrient solution for another 3 weeks growth before NaCl treatments. Defoliated stems from apex to fourth internodes were collected and immediately frozen in liquid nitrogen for RNA extraction. Three biological replicates were collected for each sample, and three plants were combined for each biological replicate.

### RNA extraction and qPCR

Samples collected from apex to 4th internode were ground to fine powder in liquid nitrogen. Total RNAs were extracted with CTAB method, treated with DNase (TaKaRa, 2270) and then purified with column from TaKaRa MiniBEST Plant RNA Extraction Kit (TaKaRa, 9769). The RNA purity and integrity were assessed by NanoDrop 2000 and Agilent 2100 Bioanalyzer. 0.5 μg total RNA was used for cDNA synthesis using HiScript II Q Select RT SuperMix for qPCR (+gDNA wiper) (Vazyme, R233–01). Vazyme-ChamQ SYBY Color qPCR Master Mix (Vazyme, Q411–02) were used for qPCR. Gene-specific primers were listed in Additional file 15: Table S9. Relative gene expression level was calculated using actin as the internal control. Three biological replicates were prepared for each time point.

### RNA-seq data analysis

High quality RNAs were submitted for mRNA sequencing library preparation and 150 bp paired-end sequencing on Hiseq X 10 platform (Illumina). Clean sequencing reads were mapped to *P. trichocarpa* v3.0 genome assembly using hisat2 [62, 63] with default parameters. The raw mapped reads for each sample were counted using htseq-count [64]. The edgeR package [65] was used to identify significantly differentially expressed genes (DEGs), with Fisher’s exact test false discovery rate (FDR) less than 0.05 as the statistical significance threshold.

### Gene co-expression network analysis

The gene expression abundance was calculated and used for weighted gene co-expression network analysis (WGCNA) [66]. The soft threshold power of the adjacency matrix for co-expression relationship between genes is 12. Hierarchical clustering was executed with a minimum module size of 300 and a cut height of 0.994. The different modules were assigned to different colors. The Weighted Correlation Network Analysis (WGCNA) included method testing the correlation between co-expression module and sample traits. The gene expression matrix of the module was computed by PCA (Principal Components Analysis) to determine the PC1, Module Eigengene (ME), which represents the module. Then, the module-trait correlation was computed using all module MEs and trait data. *P* value < 0.05 was used as the statistical significance threshold. The co-expression network was analyzed using Cytoscape [67]. Transcription factor (TF) database is derived from Plant TFDB (http://planttfdb.cbi.pku.edu.cn/index.php?sp=Ptr) [68].

### Gene ontology (GO) enrichment analysis

*P.trichocarpa* v3 Gene ontology (GO) annotation was used, and the GO enrichment was analyzed using GOstats and GSEABase packages [69] with *P*-value < 0.01. REVIGO [70] was used for visualization of enriched GO terms with default parameters.

## Additional files

Additional file 1: ** Figure S1.** Whole plant responses along the time-course of NaCl treatments. a, plants with no salt treatment; b, plants treated with 100 mM NaCl; c, plants treated with 100 mM NaCl followed with 3 days recovery, and then treated with 200 mM NaCl; d, plants treated with 200 mM NaCl directly. Photos were taken at the time points indicated on the left side. (JPG 17970 kb)

Additional file 2: ** Figure S2.** Gene cluster dendrogram based on the time-course RNA-seq data. (TIF 4128 kb)

Additional file 3: ** Figure S3.** Enriched biological pathway (BP) in green module. (PNG 206 kb)

Additional file 4: ** Figure S4.** Enriched biological pathway (BP) in turquoise module. (PNG 255 kb)

Additional file 5: ** Figure S5.** Enriched biological pathway (BP) in blue module. (PNG 301 kb)

Additional file 6: ** Figure S6.** Expression profile of representative genes from green (a), turquoise (b), and blue (c) module with RNA-seq data. (PDF 291 kb)

Additional file 7: **Table S1.** Summary of sequencing data. (XLSX 12 kb)

Additional file 8: **Table S2.** Summary of DEGs between individual time point and the corresponding control samples identified by edgeR analysis. (XLSX 9 kb)

Additional file 9: **Table S3.** DEGs between individual time point and the corresponding control samples identified by edgeR analysis. (XLSX 18432 kb)

Additional file 10: **Table S4.** Summary of co-expression modules derived from WGCNA analysis. (XLSX 9753 kb)

Additional file 11: **Table S5.** TF genes in all WGCNA modules. (XLSX 770 kb)

Additional file 12: **Table S6.** Summary of TF families in green, turquoise, and blue modules. (XLSX 11 kb)

Additional file 13: **Table S7.** Enriched GO terms of all modules. (XLSX 2113 kb)

Additional file 14: **Table S8.** Top 20 genes co-expressed with *WOX4b* and *CLE41a* from our RNA-seq data. (XLSX 11 kb)

Additional file 15: **Table S9.** Primers used for qPCR. (XLSX 11 kb)

## Data Availability

The raw sequence data reported in this paper have been deposited in the Genome Sequence Archive in BIG Data Center [71, 72] Beijing institute of Genomics (BIG), Chinese Academy of Science, under accession number CRA001067 that are publicly accessible at http://bigd.big.ac.cn/gsa.

## Abbreviations

ABA: Abscisic acid
BP: Biological pathway
BR: Brassinosteroid
CC: Cellular component
CK1: Control samples of the first salt treatment
CK2: Control samples of the second salt treatment
DEGs: Differentially expressed genes
ET: Ethylene
GA: Gibberellins
GO: Gene Ontology
H1/H3/H6/H12: 1/3/6/12 h of salt treatment
JA: Jasmonic acid
MF: Molecular function
RNA-seq: RNA sequencing
RWC: Relative water content
SA: Salicylic acid
T1: The first salt treatment
T2: The second salt treatment
TF: Transcription factor
WGCNA: Weighted gene correlation network analysis
