# Supplementary figures and images for: Genome-wide transcriptional adaptation to salt stress in Populus
Source: BMC Plant Biol. 2019 Aug 20;19:367. doi: 10.1186/s12870-019-1952-2 (PMC6701017; doi:10.1186/s12870-019-1952-2)

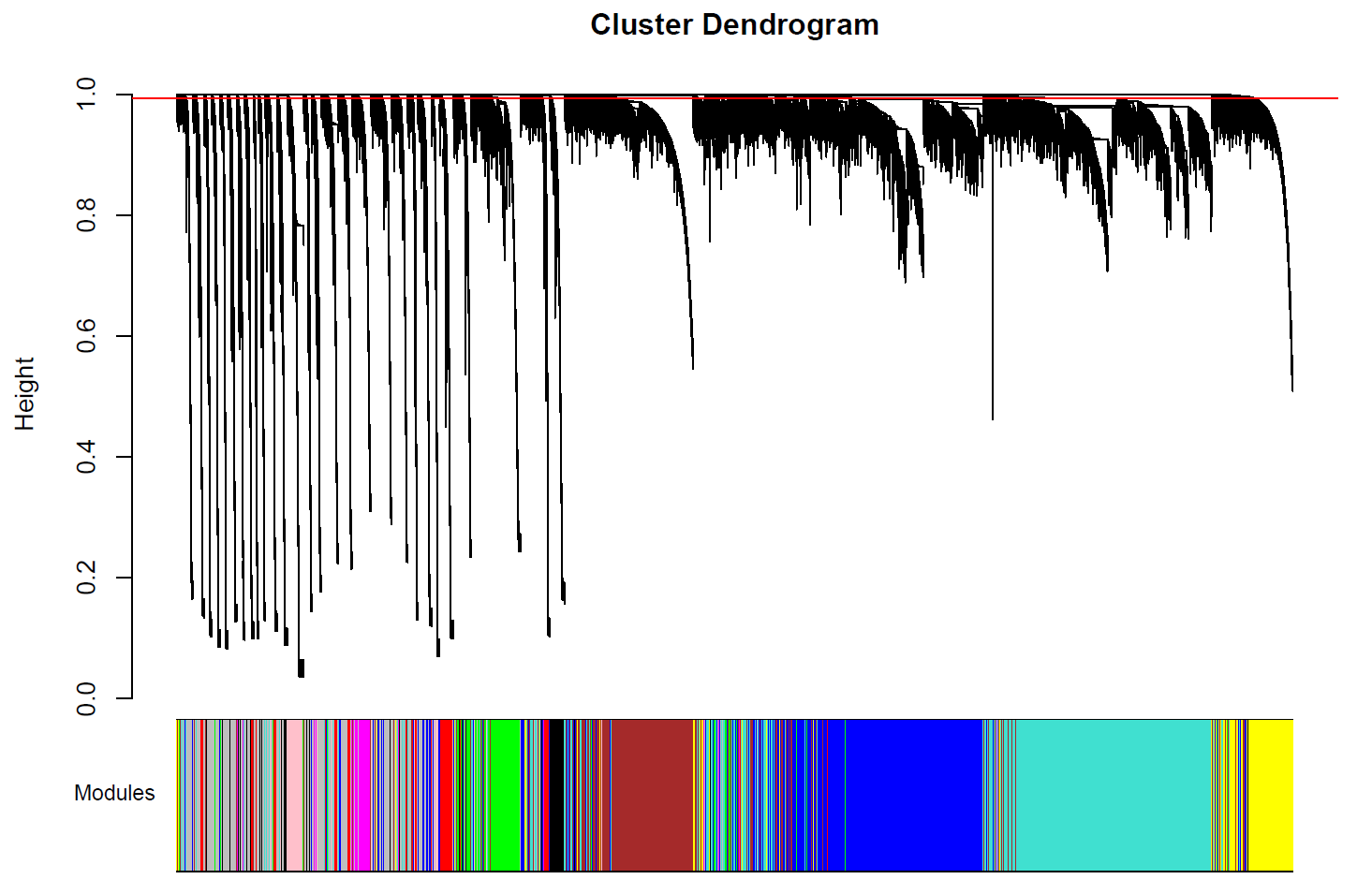

Supplement: Supplementary file 2 — Figure S2. Gene cluster dendrogram based on the time-course RNA-seq data. (TIF 4128 kb) [file 12870_2019_1952_MOESM2_ESM.tif]

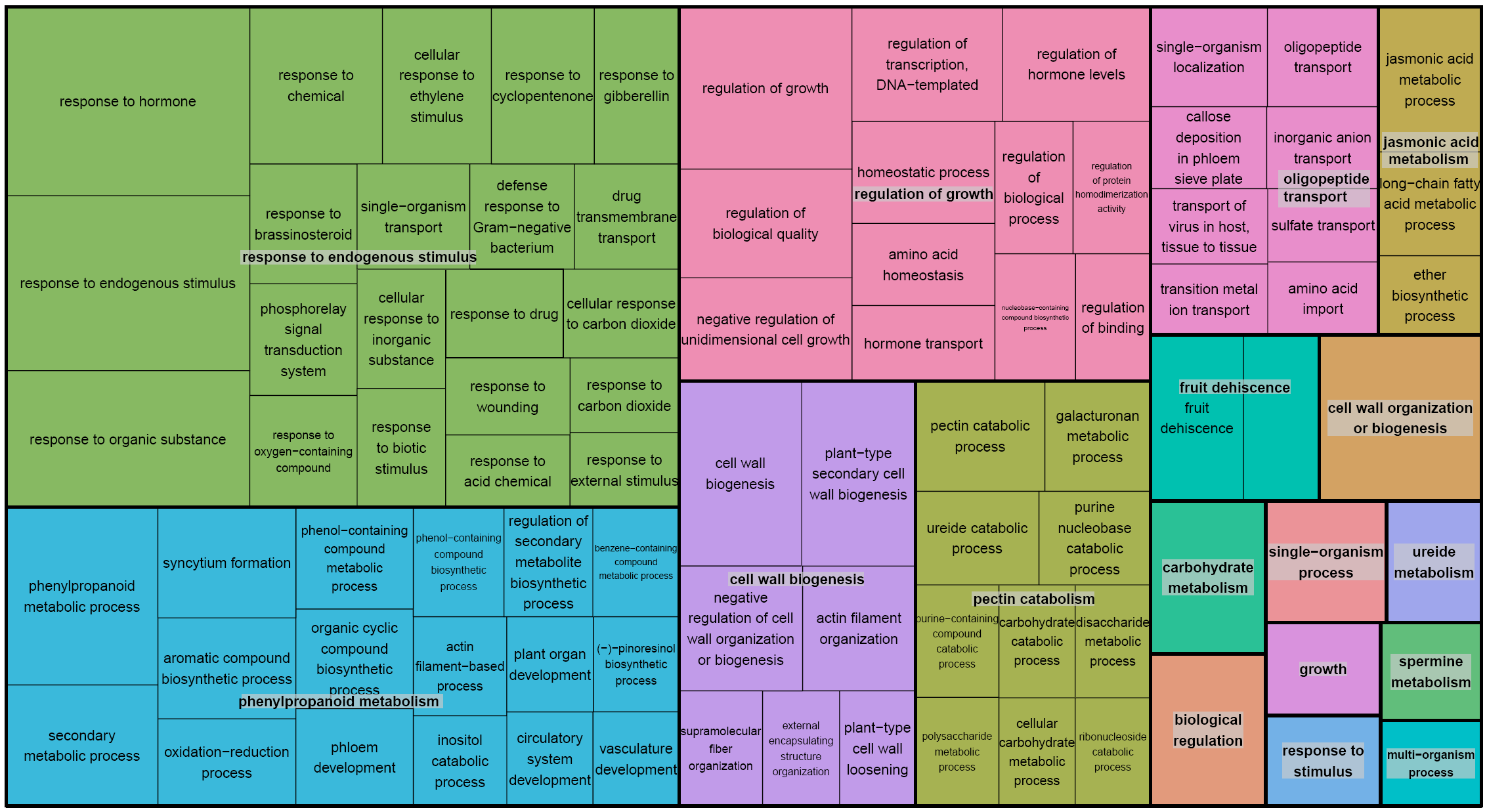

Supplement: Supplementary file 3 — Figure S3. Enriched biological pathway (BP) in green module. (PNG 206 kb) [file 12870_2019_1952_MOESM3_ESM.png]

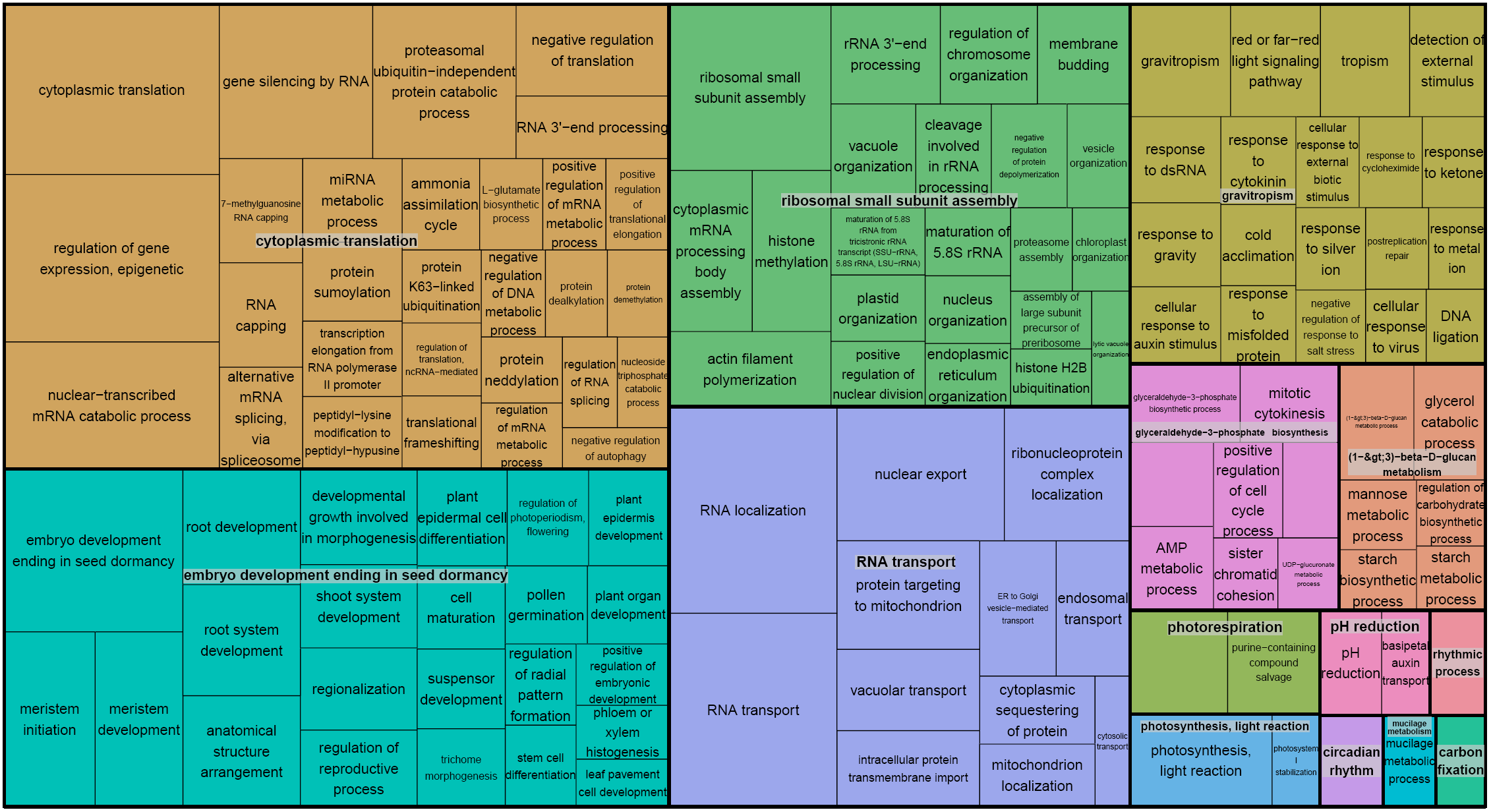

Supplement: Supplementary file 4 — Figure S4. Enriched biological pathway (BP) in turquoise module. (PNG 255 kb) [file 12870_2019_1952_MOESM4_ESM.png]

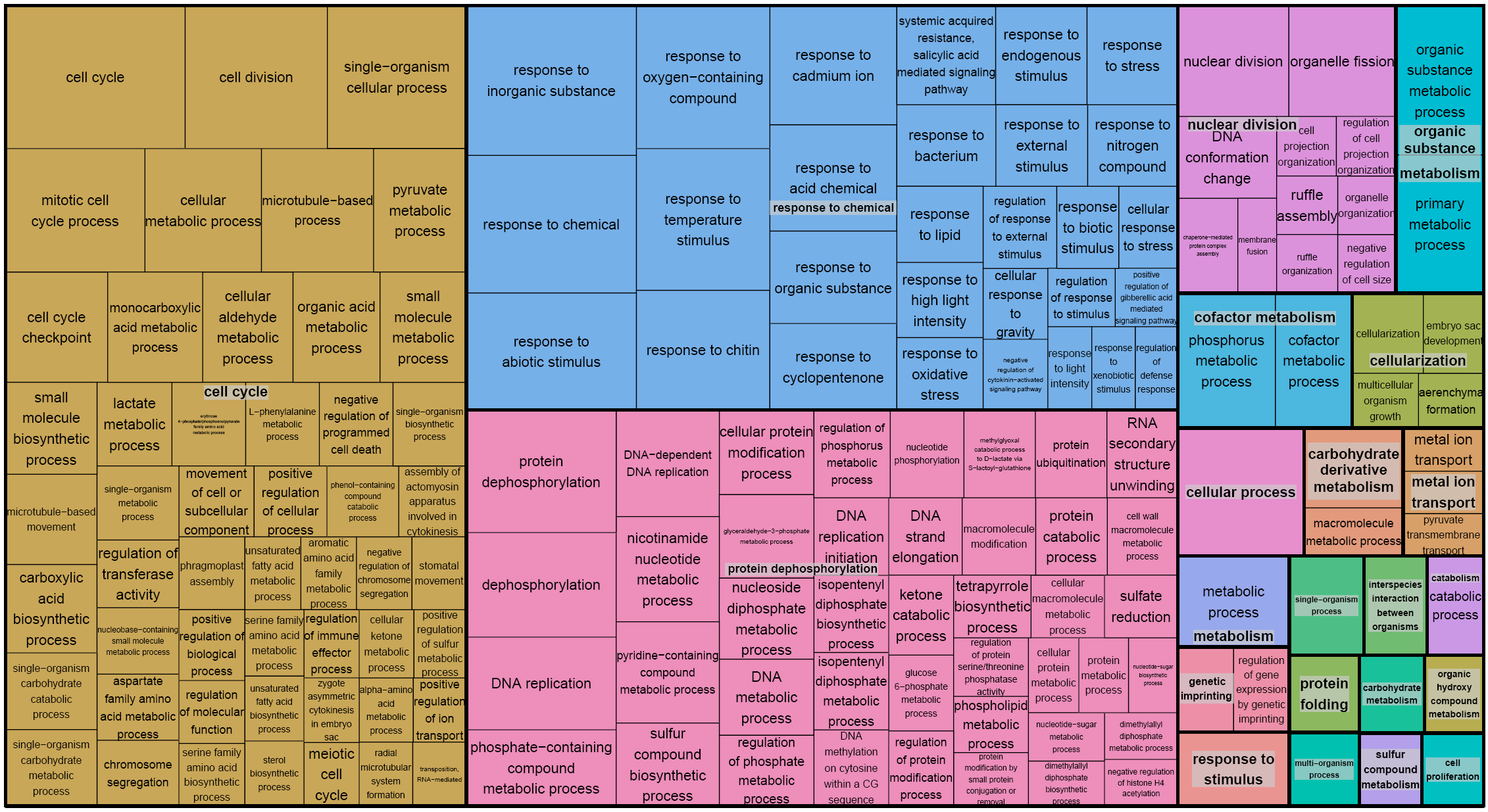

Supplement: Supplementary file 5 — Figure S5. Enriched biological pathway (BP) in blue module. (PNG 301 kb) [file 12870_2019_1952_MOESM5_ESM.png]

Gene expression (FPKM)

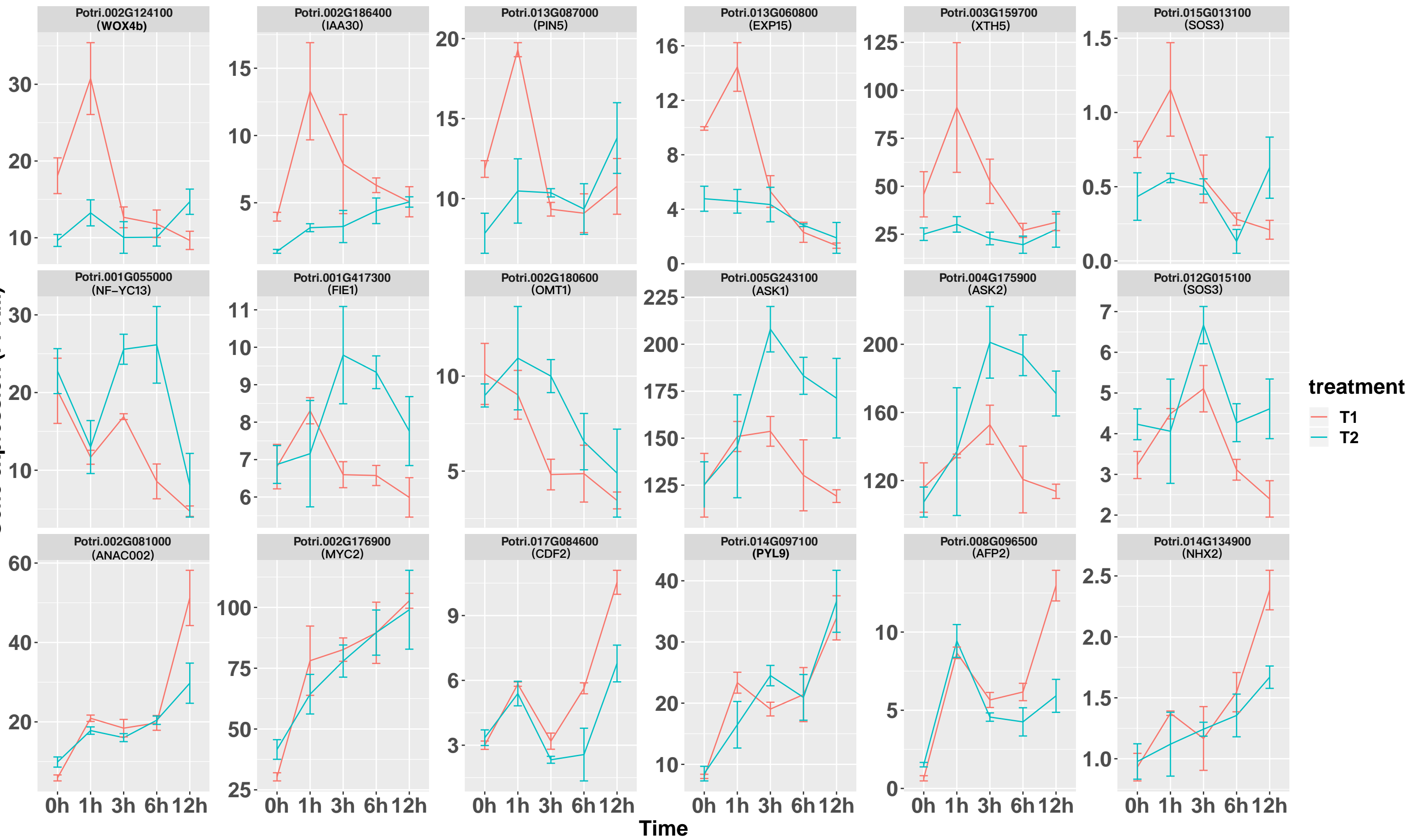

Supplement: Supplementary file 6 — Figure S6. Expression profile of representative genes from green (a), turquoise (b), and blue (c) module with RNA-seq data. (PDF 291 kb) [file 12870_2019_1952_MOESM6_ESM.pdf]
